# Hemicrania continua in a headache clinic: referral source and diagnostic delay in a series of 22 patients

**DOI:** 10.1007/s10194-012-0471-4

**Published:** 2012-07-21

**Authors:** Elisa Cortijo, Ángel L. Guerrero, Sonia Herrero, Patricia Mulero, Irene Muñoz, María I. Pedraza, María L. Peñas, Esther Rojo, Dulce Campos, Rosa Fernández

**Affiliations:** Neurology Department, Hospital Clínico Universitario, Avda Ramón y Cajal 3, 47005 Valladolid, Spain

**Keywords:** Hemicrania continua, Indomethacin, Diagnostic delay, Headache office, Referral source

## Abstract

Hemicrania continua (HC) is a unilateral and continuous primary headache with superimposed exacerbations frequently associated with autonomic features. Diagnostic criteria of HC, according to II Edition of International Classification of Headache Disorders require complete response to indomethacin. HC is probably misdiagnosed more often than other primary headaches. We aim to analyze characteristics of a series of 22 consecutive cases of HC. We recruited patients from a headache outpatient clinic in a tertiary hospital over a 3-year period (January 2008 to January 2011). We prospectively gathered demographic and nosological characteristics and considered referral source and delay between onset of headache and diagnosis of HC. Twenty-two patients (14 females, 8 males) out of 1,150, who attended the mentioned clinic during the inclusion period (1.9 %) were diagnosed with HC. All cases responded to indomethacin. No patient received a diagnosis of HC before attending our headache office. Mean latency of diagnosis was 86.1 ± 106.5 months (range 3–360). 11 patients (50 %) were referred from primary care, with 9 (40.9 %) from other neurology clinics and 2 (9.1 %) from other specialities offices. According to our series, HC is not an infrequent diagnosis in a headache outpatient clinic. Diagnostic delay is comparable to data collected in previous studies. As HC is frequently misdiagnosed, we thing there is a need for increasing the understanding of this entity, potentially responsive to indomethacin.

## Objectives

Hemicrania continua (HC) is a strictly unilateral continuous headache of moderate intensity with superimposed exacerbations often accompanied by autonomic symptoms, and absolute response to indomethacin [1–3]. HC is an uncommon primary headache disorder, so it may be misdiagnosed and mistreated. Therefore, there is a need for increasing the understanding of this entity, potentially responsive to indomethacin [4].

We aim to analyze demographic and nosological characteristics of a series of 22 new cases of HC, including reference source and latency of diagnosis.

## Methods

We prospectively evaluated consecutive new patients with HC attending a headache outpatient office in a tertiary hospital over a 3-year period (January 2008 to January 2011). In every patient, we considered age at onset, sex, background pain (side, site, type, intensity) and exacerbation characteristics (frequency, intensity, periodicity, autonomic symptoms). We collected referral source and delay between onset of the headache and HC diagnosis. Secondary headaches were excluded by magnetic resonance imaging or computerized tomography scan where appropriate. We assessed indomethacin response with a standard oral trial up to 250 mg per day, during 10 days [5]. Therapeutic results of patients in this series, including indomethacin side effects and alternative therapies have been considered in another article [6].

## Results

During the inclusion period, we diagnosed 22 patients (eight males, fourteen females) out of 1,150 (1.9 %), who attended our headache clinic with HC. All of them fulfilled ICHD-2 diagnostic criteria for HC, except the five patients without autonomic symptoms who fulfilled alternative Goadsby and Lipton criteria [7]. Mean age at onset was 41.8 ± 18.1 years (range 6–75) In all patients pain was strictly unilateral, in 14 (63.6 %) right sided and in 8 (36.4 %) exclusively left sided. Temporal pattern was always chronic and unremitting.

Background pain was generally rated as moderate intensity (5.2 ± 1.2) and exacerbations were commonly considered severe (8.4 ± 1.1) on a verbal analogical scale (0: no pain, 10: the worst imaginable pain). In our series, all patients suffered exacerbations and five (22.7 %) of them did not have associated autonomic symptoms.

All our cases responded to a standard oral trial of indomethacin, up to 250 mg per day [5]. Side effects were documented in 13 patients (59.1 %), mainly dyspepsia and dizziness. In these cases, we tried to reduce as far as possible indomethacin dose, to produce anesthetic blockade when appropriate [6], or to change to another preventative drug, mainly topiramate.

No patient had received a diagnosis of HC before attending our headache clinic. Mean latency of diagnosis was 86.1 ± 106.5 months (range 3–360). Eleven patients (50 %) were referred from primary care, with 9 (40.9 %) coming from other neurology clinics and 2 (9.1 %) from others specialities offices. No patient had received indomethacin before referral to our headache clinic.

## Discussion

HC was first designated by Sjaastad and Spierings [8] as a unilateral headache strictly responsive to indomethacin. Following this description, more than 100 cases of HC have been reported in different countries [5]. In 2004, the second Edition of International Classification of Headache Disorders (ICHD-II) included HC within “Other Primary Headache” group, and defined it as a strictly unilateral continuous headache of moderate intensity, with periodic exacerbations of variable duration and often accompanied by autonomic symptoms [1]. Bilateral or shifting-side pain localizations, or lack or enlarging of autonomic symptoms accompanying pain exacerbations can be accepted when diagnosing HC [5, 9] but, as in other series [5], we have not considered non-indomethacin responders as HC, though we will consider in the future to characterize patients with non-absolute response to indomethacin [5]. Therefore, the presence of one atypical feature, such as bilateral or shifting sides localization, can be provisionally accepted provided the rest of the features are typical.

HC is considered a predominantly female headache [5, 7, 10] and mean age at symptoms onset is around 40 years [5, 7, 11, 12]. Regarding demographic characteristics, our results are in line with previous reports.

Incidence and prevalence of HC is unknown. It was initially considered as a quite infrequent syndrome, though the increasing number of patients identified in headache offices suggested that this headache syndrome may be misdiagnosed and under recognized [4, 7, 10]. HC represents 1.9 % of headache patients attending our headache clinic, data comparable to those obtained by Rossi et al. [4] and in Vaga study of headache epidemiology [13].

Pain intensity in HC has been considered as mild to moderate when considering background pain, though reaching severe pain during exacerbations. According to ICHD-II criteria [1], patients with HC are required to have at least one cranial autonomic feature accompanying pain exacerbations, although diagnosis of HC would be possible without autonomic features when considering alternative Goadsby and Lipton criteria [7]. As in other recent series [5], a percentage of our patients did not associate autonomic symptoms.

We would like to emphasize the need for a greater awareness and understanding of HC. In a similar way as described by Rossi et al. [4], none of our patients had been diagnosed with HC and, so, they had not received indomethacin before attending our headache clinic. Latency between symptoms onset and diagnosis is larger than should be expected for an entity potentially responsive to treatment, but we found it comparable to Rossi et al.’s results [4]. Some authors have provided data that could help clinicians be more accurate in their diagnosis. Rossi et al. reported patients’ experience on medications in their case series—no patient who improved with triptans ended up having HC, but many of those with a partial response to NSAIDs or aspirin did. Cittadini et al. suggested that unilateral photophobia helped to predict HC. We have not found such predictive data in our series.

## Conclusion

Hemicrania continua is not an infrequent diagnosis in our headache outpatient clinic; burden of this entity is probably higher than observed. Diagnostic delay is high and comparable to data collected in previous studies. HC is frequently misdiagnosed and there is a need for increasing the understanding of this entity, which is potentially highly disabling for patients who can achieve a pain-free state when appropriately treated with indomethacin.
